# Differential Impact of Monsoon and Large Amplitude Internal Waves on Coral Reef Development in the Andaman Sea

**DOI:** 10.1371/journal.pone.0050207

**Published:** 2012-11-28

**Authors:** Marlene Wall, Gertraud Maria Schmidt, Pornpan Janjang, Somkiat Khokiattiwong, Claudio Richter

**Affiliations:** 1 Alfred Wegener Institute for Polar and Marine Research, Bremerhaven, Germany; 2 Phuket Marine Biological Center, Phuket, Thailand; University of Hamburg, Germany

## Abstract

The Andaman Sea and other macrotidal semi-enclosed tropical seas feature large amplitude internal waves (LAIW). Although LAIW induce strong fluctuations i.e. of temperature, pH, and nutrients, their influence on reef development is so far unknown. A better-known source of disturbance is the monsoon affecting corals due to turbulent mixing and sedimentation. Because in the Andaman Sea both, LAIW and monsoon, act from the same westerly direction their relative contribution to reef development is difficult to discern. Here, we explore the framework development in a number of offshore island locations subjected to differential LAIW- and SW-monsoon impact to address this open question. Cumulative negative temperature anomalies – a proxy for LAIW impact – explained a higher percentage of the variability in coral reef framework height, than sedimentation rates which resulted mainly from the monsoon. Temperature anomalies and sediment grain size provided the best correlation with framework height suggesting that so far neglected subsurface processes (LAIW) play a significant role in shaping coral reefs.

## Introduction

Differences in reef community and framework structure have been studied on different temporal and spatial scales and in various regions (e.g. [1], [2], [3]). These studies highlight the importance of wave action, temperature, sedimentation and other physical factors that shape coral reefs (e.g. [1], [4], [5]). Surface waves are considered a major factor affecting the light-dependent vertical zonation in coral communities (e.g. [6], [7]) but are also described to account for the horizontal differences in coral community composition between windward and leeward reef faces (e.g. [8], [9], [10]). Exceptionally strong waves following tropical storms or tropical cyclones can have devastating and long-lasting impacts on reef development (e.g. [11], [12], [13]). These episodic events along with the prevalent environmental settings determine reef appearance and growth (e.g. [5], [14]). Where reef framework development is restricted corals grow in scattered colonies or thickets attached to basement rock [3], [5], [15].

The islands in the Andaman Sea show a distinct reef development. While the ocean facing west (W) fronts of the islands do not harbor true reefs but coral communities lacking a carbonate framework, the shelf facing east (E) sides of the islands display true reefs with a high cover of corals that grow on top of a 3-dimensional carbonate framework providing a high topographic relief [16], [17], [18]. Although the region is located outside the tropical cyclone area [19], rare storms were shown to affect shallow reef areas [20]. Other episodic disturbances acting on the scale of decades or centuries are tsunamis. Although the latest (2004) tsunami was particularly severe, the subsequent reef surveys revealed only moderate coral reef damage limited to shallow water regions (above 10 m) [21] with coral recruitment and recovery proceeding rapidly in most places [22]. Given the long return periods of a disturbance of that magnitude (500 years [23], [24]) and the rareness and shallow impact of tropical storms in the area [20], the mechanical impact of tsunami and storms can be considered to affect mostly the shallow parts of the reef, where coral growth is most rapid (e.g. [25]).

A much more predictable disturbance than the exceptional tropical storm or tsunami is the seasonally recurring monsoon with heavy rains, winds and swell. In the Andaman Sea region winds peak during the SW monsoon (May - October) [26] inducing high surface waves [27]. In shallow water, breaking surface waves cause turbulent mixing and resuspension of sediments resulting in increased turbidity [28], [29]. Additionally, surface waves generate currents advecting shallow water resuspended sediments down slope (e.g. [28], [30]). As a consequence of induced sedimentation the coral community experiences reduced light intensities [28], abrasion of soft tissue [31] and smothering of corals by sediment [31], [32].

An underexplored but potentially important source of disturbance in coral reefs is the impact by internal waves [33]. In contrast to surface waves, internal waves travel along density gradients within the water column and may attain much larger amplitudes [34]. They are common in the world oceans (e.g. [35], [36], [37], [38]) and in the Andaman Sea they show extraordinary large amplitudes of 60 m or higher and travel with speeds of up to 2 m s^−1^ [39], [40]. In shoaling water, LAIW may steepen, break and form bores, which travel upslope and mix with surface water [41], [42], [43]. Coral reefs subjected to LAIW are confronted with rapid and strong fluctuations in temperature, pH, oxygen and nutrients [16], [35], [41], [44]. Previous studies in different regions showed mixed effects of internal waves on coral reefs [45], [46], likely due to regional differences in frequency and magnitude of the disturbance. Frequent and severe LAIW, associated with temperature drops of up to 10°C at subtidal frequencies are likely detrimental and may account in part for the marked differences in coral reef development on opposite sides of offshore islands [35].

In the Andaman Sea, LAIW and surface waves both impinge from westerly directions, but vary in intensity between seasons: LAIW peak during the dry season (NE monsoon, November – April) [16], [27], when the water is well stratified and the thermocline/pycnocline is shallow [47]. Surface waves are largest during the wet season (SW-monsoon May – October) when southwesterly winds are strongest. Although previous studies in the Andaman Sea highlighted pronounced differences in coral nutritional status, coral community structure and reef framework development between the exposed (W) and sheltered (E) faces of the Similan Islands, the limited spatial extent of these investigations (100 s–1000 s of m, [16], [46], [48]), along with the spatial covariance of the environmental factors, made it difficult to assess the relative contribution of monsoon and LAIW to the observed biological changes.

The present study attempts to disentangle the effects of LAIW and SW-monsoon on shallow water coral reefs. It also extends the limited spatial scale of previous studies to the scale of the continental shelf (10 s of km), taking advantage of the geographical setting of the Thai shelf to investigate if and to what extent LAIW and/or monsoon govern the coral reef development in the Andaman Sea. The study area features a string of islands between the shelf break and Thai mainland and, hence, differential cross-shelf gradients of LAIW and monsoon impact: while LAIW, with long wavelengths relative to bottom depth, are expected to dissipate part of their energy in shoaling water, resulting in a cross-shelf decrease in LAIW intensity [33], monsoon waves are not expected to attenuate across the Thai shelf. They are much shorter relative to 70 m at the outer and 50 m in the inner shelf and propagate unattenuated in the cross-shelf direction. As a result, the islands on the Thai shelf are exposed to a differential impact of LAIW and monsoon across the shelf, where LAIW intensity varies much more markedly across the shelf than monsoon impact.

## Materials and Methods

### Sites

**Figure 1:**
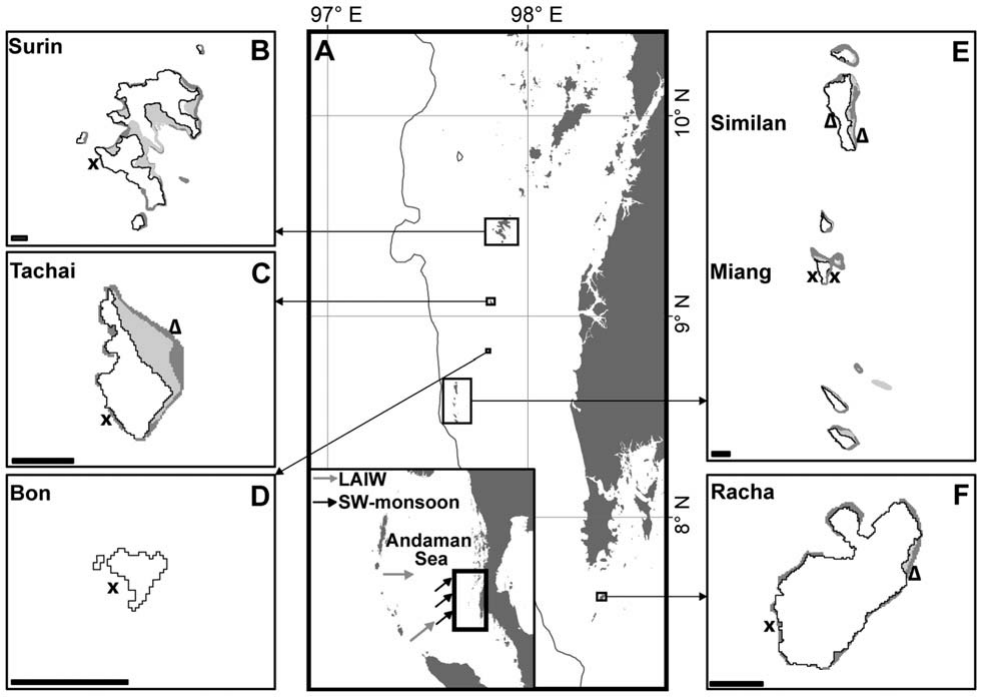
Location of sample sites, Andaman Sea, Thailand. **A**) Map of the study area in the Andaman Sea off-shore the west coast of Thailand with inset showing the Andaman Sea from the Nicobar Islands Arc to the southeast Asian mainland of Burma and Thailand (Mainland: Wessel and Smith [79], Bathymetry: Smith and Sandwell [80]). B–F) Close-up maps of the study islands (Surin, Tachai, Bon, Similan, Miang and Racha) with sampling sites (UNEP Coral Millennium Project). Crosses: core sampling sites with high-resolution temperature monitoring and framework height determination (at Miang W and Surin W additional CTDs were deployed) ; triangles: sites where only framework height was measured (Tachai E and Racha E the present study, Similan E and W framework height derives from [16]). Scale bar represents 1 km.

Five islands located in the Andaman Sea on the continental shelf off the western Thai coast were chosen for this study. From north to south the study islands are: Surin, Tachai, Bon, Similan, Miang and Racha (Fig. 1), which differ in their distance to the shelf break: 62 km, 43 km, 35 km, 22 km (Similan and Miang) and 53 km, respectively. Six sites were selected for both environmental monitoring and framework measurements with 5 of them facing W, thus being exposed to LAIW and SW-monsoon impact and 1 site was chosen as a control site located on the LAIW- and SW-monsoon sheltered E coast of Miang (Fig. 1; Surin W, Tachai W, Bon W, Miang W, Miang E and Racha W; these sites represent the core sampling sites). Framework height was further determined on 4 additional sites (Tachai E and Racha E; the present study, Similan E and W derived from Schmidt et al. [16], Fig.1). Site selection was done by snorkelling along the coast of the selected islands. The sites with the most vigorous reef development were chosen to compare the maximum reef development potential between sites. The depth for all surveys and deployments was 15 m. The study was performed within 9 cruises during both, dry and wet seasons from November 2009 through November 2011 (Table S1).

### Reef framework height determination

The lack of a true carbonate framework in the exposed faces of the islands [16] precluded the use of coring to determine the height of the reef framework (e.g. [49]). Reef framework height was determined according to Schmidt et al. [16]. Scaled high-resolution (14.7 Mpix) photographs were taken with a digital camera (Canon G10 camera with a 28 mm wide-angle lens in a Canon WP-DC 28 housing), showing the elevation of coral framework above its basement (sediment or rock). A measuring stick with 5 cm tick marks positioned perpendicular to the bottom at the study depth in front of the framework was used as a reference. Up to 45 images were taken along and at both sides of a 50 m transect where corals or carbonate structures were present. The height of the framework was calculated by image analysis with the software ImageJ as the closest distance (in cm) between the top of corals/carbonate structure and the basement. Only corals/carbonate structures in the same plane as the stick were measured (corals/carbonate structures in the front of or behind of the measuring stick would have been over or underestimated in height). This resulted in 1 to 5 measurements per image. A total of 268 framework measurements was carried out (in average 38 per site, with a minimum of 21 in Racha E and a maximum of 45 in Miang W and Bon W). Framework height data for Similan E and W derived from Schmidt et al. [16].

### Environmental characterization

#### Temperature

Temperature was monitored with temperature loggers (3 Onset HOBO Tidbits per site, temperature resolution: ± 0.2°C) deployed approx. 30 cm above the bottom fixed on the sediment trap holders. Temperature was recorded at the core sample sites (Fig. 1; temperature data for Similan E and W were set equal to Miang E and W, respectively; justification see Schmidt et al. [16]). The limited storage capacity of the loggers and limited access to the study area during the monsoon period (May- to October) required the combination of the data of 2 adjacent loggers (sampling interval: 6 minutes, phase lag between loggers: 3 minutes) to 1 time series yielding a composite sampling interval of 3 minutes. During the remainder of the year, where access to the study area was not limited, the loggers were programmed to sample every 3 minutes and exchanged more often. The loggers were calibrated in a water bath at room temperature in the lab with a high precision temperature meter (Amarell ama-digit ad3000th, ± 0.01°C, off-sets ranged from 0.33 to −0.24°C).

#### Conductivity-Temperature-Depth (CTD) – Profilers

In December 2010 and March 2010 2 Seabird CTDs (Seacat SBE 19plus and 19plus V2) were deployed at 2 sites (Miang W and Surin W) to record temperature, conductivity and oxygen or pH (19plus V2 only). Instruments were deployed for approximately 48 h in December (both sites) and 5 and 14 days in March (Surin W and Miang W, respectively) and the logging interval was set to 5 minutes.

#### Sedimentation rates

At all core sampling sites (see Fig. 1) 3 sediment traps were deployed in a distance of 12 m to each other. Each of these traps contained 2 glass tubes sealed with a bottom lid (Ø 60×180 mm; 1∶3), which were mounted on a steel rod with concrete base. The bottle openings were placed 50 cm above ground and were fitted with a baffle (grid size 1 cm) to minimize hydrodynamic disturbances in the collector and to prevent the entrance of larger organisms [50]. The traps were deployed in November/December 2009 and exchanged 6 times during the research period until November 2011 (Table S1). From sediment samples macroinvertebrates were removed and the samples dried at 80°C for 24 h to constant weight. The sedimentation rate was calculated in mg dry mass cm^−2^ day^−1^.

#### Grain size distribution

Close to but beyond the hydrodynamic influence of each sediment trap 1 sediment core (in total 3 cores per site, Ø 5 cm approximately 5 cm into the sediment) was collected in January, March, May, July, December 2010 and March 2011 (Table S1). Each core sample was mixed to assure homogeneity and a subsample of 20 g was mixed with 200 ml distilled water and 8 ml sodium hexametaphosphate and stored over night at room temperature. The sediment sample was then poured into a shaker with 63, 125, 250, 500, 1000, and 2000 µm mesh size. Shaking was performed for 5 minutes, the particles of each mesh size washed into preweighed aluminum cups and dried over night at 80°C for dry weight determination. Grain size values of each site are expressed as dimensionless mean phi value [51] of the 3 sediment cores, and classified according to the Udden-Wentworth [52] size scale into coarse or medium sand (grain size phi: 0–1 coarse sand, 1–2 medium sand and >2 fine sand).

### Data processing and statistical analysis

Statistical tests were performed with the programme R, Sigma Plot v11 was used for plotting the data.

Normality and homoscedasticity of the data was ascertained with the Shapiro-Wilk and Fligner-Killeen test, respectively. Non-parametric tests were applied when data did not comply with the parametric test requirements of normality and homogeneity of variances. Differences between seasons and sites were tested with ANOVA and Tukey HSD post-hoc analysis, Kruskal-Wallis rank sum test and the Wilcoxon rank sum test, accordingly. Spearman's rank correlation was used to explore the correlations of pH, oxygen and conductivity with temperature.

#### Environmental variability characterization

To assesses the differential impact and the relative contribution of the 2 disturbance regimes, LAIW and monsoon, environmental parameters were calculated characterizing changes in environmental conditions.

#### Parameter characterizing LAIW

Temperature anomalies: Abrupt and pronounced sea-bed temperature drops are a hallmark of internal waves diapycnal mixing in coastal water, as opposed to the gradual change induced by changing horizontal currents. The integration of negative temperature deviations from the mode (i.e. the most common value) temperature is thus a good measure of the intensity of environmental fluctuations. The temperature anomalies were calculated as cumulative degree days (DD) for each month according to Schmidt et al. [16] (a modification of Leichter and Genovese [45] with the only differences that residuals were calculated from the daily running mode instead of mean). Temperature values were subtracted from the daily running mode, this negative deviations (°C) multiplied with the sampling interval (in days) and summed up for each month. To quantify the yearly LAIW impact (yearly mean values) cumulative DD per month were averaged for an entire year (December 2009 to November 2010). Additionally, a measure for the period of maximum temperature variations (maximum impact) was calculated using only the average of the cumulative DD per month of the LAIW-intense dry season (December – to May 2010, as during this time LAIW were strongest).

#### Parameters describing monsoon

As wave measurements during SW-monsoon seasons were not feasible for logistical reasons we relied on sediment parameters known to reflect hydrodynamic conditions i.e. surfaces waves and currents [26], [53]. To quantify the intensity of turbulences due to both LAIW and SW-monsoon the amount of sediment collected in the traps (sedimentation rate) as well as bottom sediment characteristics (bottom sediment grain size) were used as proxies.

Sedimentation rate: The sediment captured by the traps derived from and reflected the hydrodynamic conditions at site, such as (1) currents scouring the sea-bed and enhancing the particle load in the bottom boundary layer [28], [54], (2) oscillating currents due to ocean swell and surface waves [55] and (3) LAIW leading to resuspension and subsequent sedimentation of bed-load particles [33]. Because there are no rivers close to the study area run-off and major terrigenous sediment input can be neglected. Sedimentation was thus a composite annual proxy for monsoon and LAIW, where the surface wave impact was captured during the SW-monsoon sampling period (May-October), while the internal wave impact is reflected in the dry season samplings (November – April).

Grain size phi: In a previous study [26] in Thailand bottom sediment properties were used as a proxy for reef exposure to rank-order sites. This proxy was adopted in the present study and slightly modified. Because all sites were classified and grouped as high energy reefs according to the scale of Scoffin et al. [26] (almost mud-free reefs - mud content <63 µm: 2.40–4.64% weight; Kruskal-Wallis rank sum test: χ2 = 5.68, df = 5, p-value = 0.338; Table S2). Thus, we used the mean grain size phi values to quantify the hydrodynamic energy for each site and differentiate between sites. Average yearly hydrodynamic conditions were derived by averaging all grain size phi values per sites. The maximum impact was anticipated to occur during the wet season when the SW-monsoon prevails and hence, values from the July 2010 sampling were used.

These parameters allowed relating them to the reef framework height at site and comparing sites. General linear models were fitted to the data with reef framework height as dependent and the environmental parameters as independent variables.

## Results

### Reef framework

**Figure 2:**
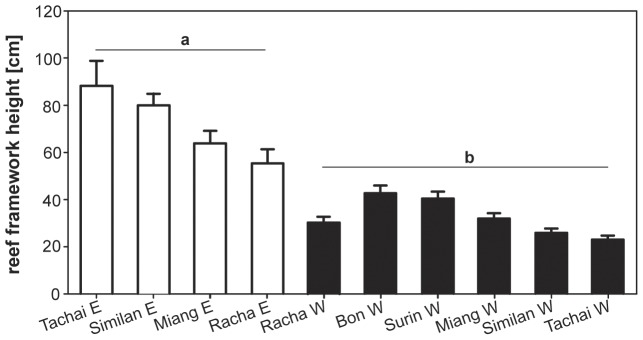
Reef framework height above bottom. Reef framework height (mean ± SE) measured at all study sites. Open bars are E sites (a), black bars W sites (b), showing significant differences (Welch's t-test: t = 10.66, df = 131, p<0.001). Framework height is arranged from left to right with lowest to strongest LAIW (large amplitude internal waves) intensity (intensities were estimated for sites where no temperature record were available (Tachai E, Racha E)).

The LAIW- and monsoon-exposed W sides of Tachai, Similan, Miang and Racha showed a significantly lower framework development than the corresponding E sides protected from LAIW and monsoon (Welch's t-test: t = 10.66, df = 131, p<0.001; Fig. 2).

### Temperature variability

**Figure 3:**
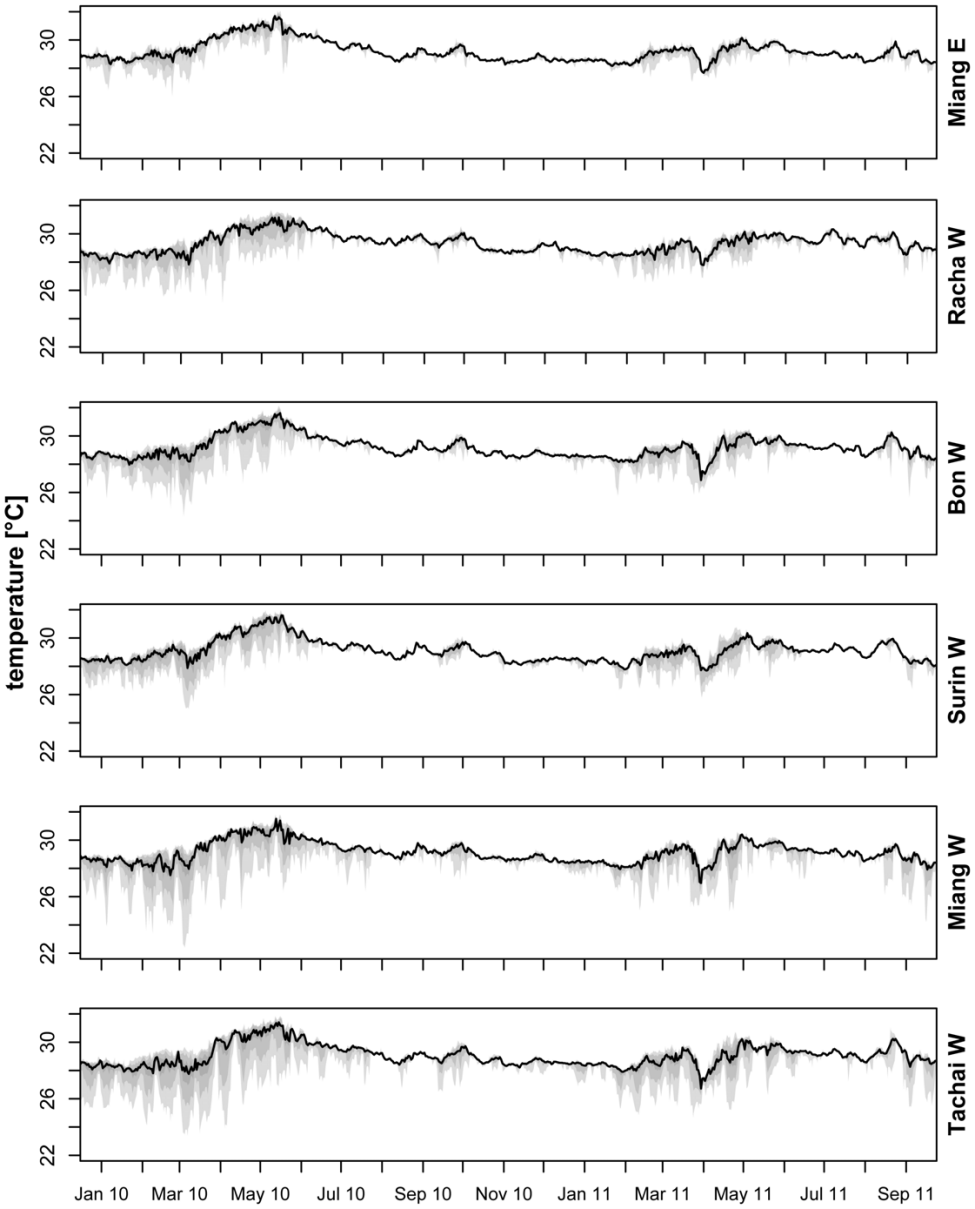
Sea bed temperature at 15 m. Temperature record for the 6 core sampling sites (cf. Fig. 1) showing median (black lines) and quantil ranges in shades of grey (light gray: 0–1, intermediate gray: 0.05–0.95 and dark grey: 0.25–0.75). Sites are arranged from top to bottom (Miang E, Racha W, Bon W, Surin W, Miang W and Tachai W) with lowest to strongest LAIW (large amplitude internal waves) intensity.

**Figure 4:**
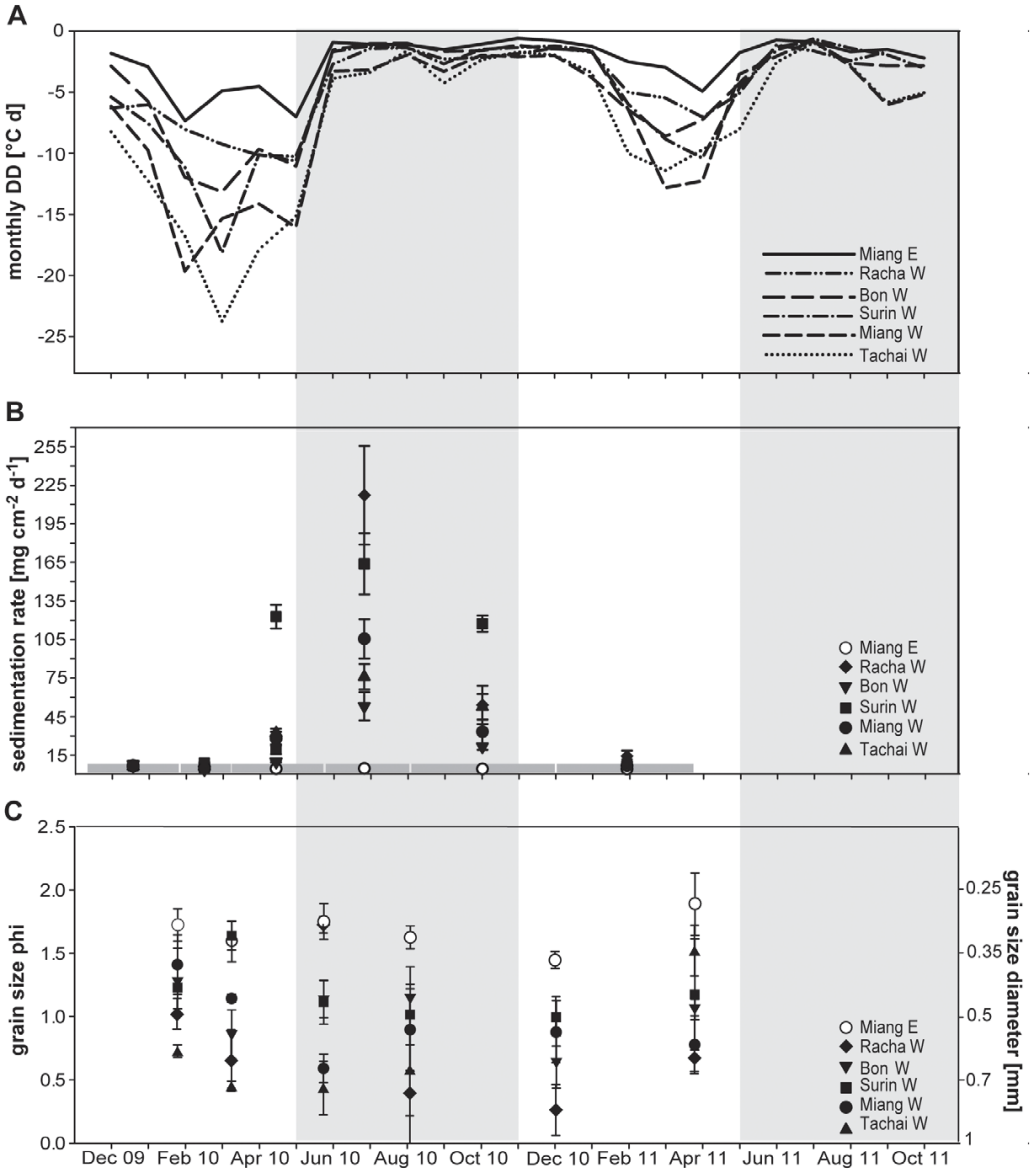
Negative temperature anomaly, sedimentation rate and grain size variation. All parameters are displayed for the study period November 2009 to December 2011 for the core sampling sites (cf. Fig. 1). A) Negative temperature anomalies calculated as cumulative degree days (DD) per month. B) Temporal evolution of sedimentation rates (mean ± SE) as dry sediment mass per cm^2^. Deployment periods are indicated by the grey broken line parallel to the time axis. C) Grain size phi (mean ± SE) or diameter (mean ± SE [mm]). Grey shaded area corresponds to the SW-monsoon season May-November.

The temperature records at the 6 core sampling sites displayed differences in the intensity and frequency of temperature anomalies throughout the year, between sites and years (2010 vs. 2011 dry season, Fig. 3). On the basis of temperature fluctuations 2 seasons could be distinguished (Fig. 4A) with a higher frequency and intensity of temperature variation during the NE monsoon (November to April) compared to the SW monsoon season (May to October) (t-test: p<0.0085, Table S3 for p-values per site). Negative temperature anomalies were visible at all sites and these anomalies calculated as monthly cumulative DD differed between sites (ANOVA, p<0.001, Table S4) with higher temperature fluctuations on W than on E. Not all W sites showed significantly different temperature variations compared to Miang E suggesting LAIW attenuation, which however did not seem to be related to distance from the shelf break (Spearman rank correlation of yearly mean monthly cumulative DD and distance to the shelf break, ρ = 0.3, S = 14, p = 0.683). Additionally, between year's temperature anomaly intensity and frequency differed with stronger LAIW forcing in 2010 compared to 2011 (Fig. 3).

### Physico-chemical variability with temperature

Changes in chemical water parameters (conductivity, oxygen and pH) were well correlated with temperature fluctuations at the most distant and closest site to the shelf break (Surin W and Miang W, respectively). All correlations were highly significant and showed a decrease in pH, oxygen and an increase in conductivity with decreasing temperature values (Table 1).

**Table 1:** Spearman's rank correlation analyses of conductivity, pH and oxygen with temperature derived from CTD records at Miang W and Surin W (cf. Fig. 1) during December 2009 and March 2010 deployments.

sites	season	correlated parameter		r	p	n
Miang W	December	temperature	conductivity	0.99	***	289
	December	temperature	pH	0.43	***	
	March	temperature	conductivity	0.92	***	4609
	March	temperature	oxygen	0.74	***	
Surin W	December	temperature	conductivity	0.86	***	577
	December	temperature	oxygen	0.65	***	
	March	temperature	conductivity	0.99	***	1008
	March	temperature	pH	0.78	***	

(ρ = correlation coefficient, n = number of measurements, p = probability level, significance levels are: * p<0.05, ** p<0.01, *** p<0.001).

### Sedimentation rates and bottom sediment grain size

Clear seasonal differences in sedimentation rates were measured with largest values at the height of the SW monsoon (Fig. 4B). During the dry season sedimentation rates were low and similar between sites (Kruskal-Wallis test: χ2 = 7.6246, df = 5, p-value = 0.1782). Around May rates started to increase only for the island W sites (Fig. 4B; Kruskal-Wallis test: χ2 = 23.8949, df = 5, p-value<0.001).

Benthic sediment was composed of coarse to medium sand (Fig. 4C). In the W bottom sediments were coarser than in the E (ANOVA, p<0.001, Table S5) with the only exception found for Surin W with less coarse sediment (post-hoc Tukey HSD pair wise comparison Surin W and Miang E: p = 0.891). Racha W showed a strong fluctuation in grain size through out the year with comparable fine sediment in May and the coarsest sediment in July (Fig. 4C).

### Reef framework and environmental indices

**Figure 5:**
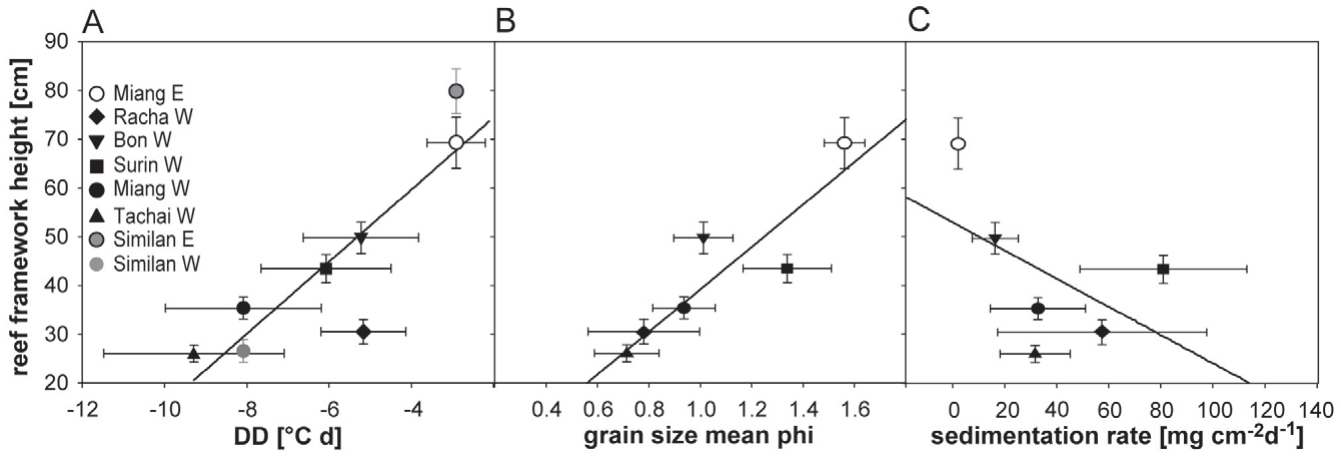
Reef framework height as a function of environmental parameters. Reef framework displayed as a function of A) temperature anomaly (DD = degree days), B) grain size phi and C) sedimentation rate. Environmental parameters represent mean values for an entire year - 12.2009–12.2010 derived for the core sampling sites (cf. Fig. 1): A–C) Miang W, Miang E, Bon W, Tachai W, Surin W, Racha W and for A) additionally from Similan E and W [16]. Temperature anomaly values for Similan W and E were set equal to Miang E and W, respectively; justification see [16]. All values are given as mean ± SE.

Both the environmental parameters and reef framework height displayed differences between sites but these differences were not always statistically resolvable. A general linear model was calculated with reef framework height as dependent variable and the different environmental indices derived for each site as independent variables. The results indicate that both temperature anomaly and grain size explain the reef framework height (core sampling sites: (1) temperature anomaly: yearly mean values n = 6, r^2^ = 0.68, p = 0.045 (Fig. 5A), and (2) grain size: yearly mean values: n = 6, r^2^ = 0.81, p = 0.015 (Fig. 5B); Table S6). The addition of two further sites from the study Schmidt et al. [16] with available temperature and framework information (see section 2.3) to the model supported and even increased the relationship between framework height and temperature anomaly (yearly mean values: n = 8, r^2^ = 0.77, p = 0.004, Table S6, Fig. 5A). Framework development was inversely related to degree days and grain size. In contrast, the sedimentation rate did not follow the reef framework pattern (yearly mean values: n = 6, r^2^ = 0.27, p = 0.286, Table S6, Fig. 5C) with highest sedimentation rates not corresponding to lowest reef framework height. A multiple linear model best explained the reef framework height (for core sampling sites only: n = 6, r^2^ = 0.97, p = 0.004) and resulted in an improvement of the relationship between reef framework height and each single predictor when maximum impact of temperature anomaly and grain size (p = 0.029, p = 0.009, respectively) were combined. This indicates that the 2 disturbances with anticipated maximum impact during different seasons best explained the reef formation.

**Figure 6:**
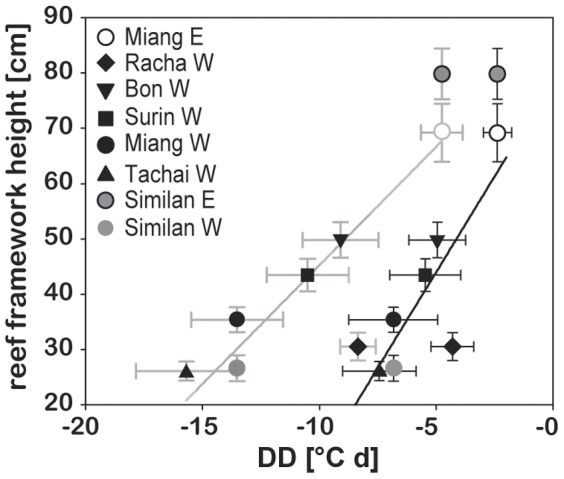
Reef framework height versus 2010 and 2011 dry season negative temperature anomaly. Reef framework height plotted as a function of dry season temperature anomaly (DD = degree days) reflecting the negative temperature anomalies experienced during the peak large amplitude internal waves (LAIW) period for 2010 (grey error bars and regression line) and 2011 (black error bars and regression line) for core sampling sites (cf. Fig. 1) plus Similan E and W (temperature anomaly values were set equal to Miang E and W, respectively; justification see Schmidt et al. [16]). All values are given as mean ± SE.

The length of the temperature records (>2 years) allowed us to resolve the cumulative DD for 2 consecutive dry seasons and relate temperature anomalies for 2011 to reef framework height. The LAIW index for the dry season revealed a consistent order of the islands according to the intensity of temperature fluctuations between subsequent years (Fig. 6; period of max impact 2011: n = 8, r^2^ = 0.77, p = 0.004; Table S6).

## Discussion

To the best of our knowledge this is the first study to explore the relative roles of internal wave and monsoon exposure on coral reef development. We could show that across the entire shelf, temperature variability showed a clear correlation with reef framework height explaining a high percentage of the variability in framework development (77%, p = 0.004). The reef framework height was not satisfactorily explained by the monsoon in terms of sedimentation rates, however correlated fairly well with the grain size of the bottom sediments. Temperature anomalies and bottom sediment grain size together provided the best correlation with framework height, which suggests a combined impact of LAIW and monsoon waves. Although data on the vertical distribution of temperature and sedimentation rate are lacking, a previous study on the vertical distribution of temperature variability and framework development in the Similan Islands showed a direct relationship between LAIW intensity and depth. The authors hypothesized that LAIW might be responsible for the reduced framework in deeper reef areas [16]. The here established relationship between LAIW intensity and framework height supports their assumption. Given that surface wave impact is inversely related to depth [56], LAIW and monsoon affect opposite ends of the vertical scale, with LAIW shaping the deeper and monsoon the shallower parts of the reef.

### Large amplitude internal waves

Our findings suggest that strong LAIW-induced diapycnal mixing suppresses reef development. This is in line with Benzoni et al. [57] who observed an inverse relationship between framework development and upwelling intensity in the Gulf of Aden. Also the reduction in coral abundance and height observed by Schmidt et al. [16] on LAIW-exposed Andaman Sea reefs are reminiscent of the scattered distribution of colonies near upwelling areas [58], [59].

Previous findings in the Andaman Sea near the shelf break showed that changes in temperature along the Similan Islands co-varied with pH, oxygen, nutrient and salinity [16]. The present study allowed tracing similar correlations to the most distant site to the shelf break (Surin W) suggesting that LAIW do not necessarily dissipate near the shelf break but continue to propagate shoreward onto the shelf. Observational and modeling work has shown that LAIW can advance onto the continental shelf [60], [61] even though they have their largest amplitude in deep offshore waters (150 m) [39]. Interestingly, the degree of variability observed at our study sites did not relate in all cases to cross-shelf distance from the shelf break, in line with models suggesting that bottom topography can result in refraction and focusing of LAIW wave energy [60].

The available studies on internal waves [35], [42], [45] and coastal upwelling on coral reefs [59], [62] are consistent with our earlier notion that diapycnal mixing is a mixed blessing. While the supply of nutrients and plankton are regarded as beneficial [44], [46], [63], the cold temperatures (e.g. [64], [65], [66]) and low pH (e.g. [67], [68]) can be stressful for corals. Positive, negative and synergistic effects are a matter of the intensity of diapycnal mixing and may vary also between species. Although some earlier studies suggested that diapycnal mixing may affect the lower reaches (below 50 m [35]) of the reef slope (due to LAIW), or the reef's cementation (due to upwelling) [67], our study highlights the importance of diapycnal mixing for reef development in shallow water for several continental shelf islands in the Andaman Sea. Considering the ubiquitous occurrence of LAIW in the world ocean [40] they potentially shape coral reefs in other areas.

### SW-monsoon – bottom sediment grain size

The reef framework height also correlated with grain size composition. Scoffin et al. [26] pointed out the importance of hydrodynamic processes for the linear extension rate and skeletal density in *Porites lutea* where linear extension rates were found to be inversely related to local hydrodynamics. A relationship between coral community structure and grain size was also observed for Hawaiian reefs [4]. Grain size distribution is a function of wave action, currents and swell, but also determined by i.e. bathymetry [69] affecting sediment mobilization and deposition [55]. Although it has been shown that during the dry season LAIW-induced strong currents may account for resuspension and upslope transport of sediments [33] much of the resuspension is due to surface wave resuspension in shallow water with subsequent down slope transport of sediments [55], [70]. In general, the grain size properties of the study sites showed that W reefs were subjected to higher current and wave action during both seasons with slightly increased values during the peak SW-monsoon season (Fig. 4C). This suggests that during the NE monsoon LAIW-induced currents lead to an upslope transport and accumulation of finer sediment and to a transport of fine sediments out of the study area during SW monsoon.

### SW-monsoon– sedimentation rate

From a climatological point of view, the reversal, onset and extent of the Asian monsoon is well established [71] but a lack of information exists about the oceanographic conditions [72], in particular of wave height and period. While the NE-monsoon is generally characterized by low wind conditions the SW monsoon is the windy season reaching its full extent during July to September with winds speeds of up to 10 m s^−1^ [26], [27]. Strong wind conditions generally prevailed for several days followed by a period of calm conditions [73]. Sedimentation rates in our study clearly displayed a monsoon signal with peak rates during June through August during the height of the SW monsoon [26], [71]. Sedimentation on corals is known to be detrimental [74], [75], [76]. The degree of damage, however, is species-specific, a matter of duration and of the origin of the sediments with terrigenous silty sediments exerting a stronger impact than carbonate sands [74], [75]. A long-term study showed that sedimentation caused bleaching, necrosis and partial mortality in corals [76]. It is thus plausible that the coral community on the exposed W sites suffered from increased sediment load both directly and indirectly (due to lower light levels). During the intermittent calm periods during the SW monsoon and during the NE monsoon, however, release of sedimentation stress may have led to recovery in the corals. Higher survival rates during experimentally excluded light conditions, higher photosynthetic performance and the increased energy status of W corals [46], [48] suggest that their higher protein and pigment content enables them to cope with harsh peak SW monsoon conditions particularly with the reduced light conditions. The fact that sedimentation cannot explain reef framework height agrees with findings of Bak and Meesters [77] who did not observe a correlation between reef development and sedimentation.

### Other factors

Biological factors i.a. larval supply, settlement space, or bioerosion can also restrict reef formation. Previous investigations showed that settlement space is not limited in the Similan islands [16] or elsewhere on the Thai shelf (Phongsuwan, pers. comm.). Data on larval recruitment rates only exist for the Similan Islands and where shown to be higher in W than in E but survival rates of juvenile corals to adult stage were lower [78]. This finding of a lowered survival rate complies with the stressful conditions exerted by LAIW and SW monsoon and the observed reduced framework. Likewise, diapycnal mixing was described to enhance the establishment of an internal bioeroder community and combined with a reduced cementation [67] favors bioersion and supports the observed relationship between LAIW intensity and framework formation.

We did not investigate to what extent the differences in coral community composition accounts for the observed differences in framework development. Yet as shown by Benzoni et al. [57] the potential to develop a reef framework is not a matter of community dominance by growth form or genus.

### In summary

Although a plethora of disturbances are known to inhibit the development of coral reef framework (i.a. wave impact, upwelling, strong temperature fluctuation, turbidity), with a highlight on surface waves [6], [7], our study is the first to show internal waves may rival the importance of surface waves in shaping reef communities. Because LAIW are not restricted to the Andaman Sea but common [40] in many other coral reef regions, they may play an important yet unexplored role in the reef building process.

## Supporting Information

Table S1 **Cruise schedules and tasks.** Time table for temperature logger, sediment trap and CTD deployments/exchanges at the core sampling sites (cf. Fig. 1).
(DOCX)

Table S2 **Comparison of mud content (%) of bottom sediment samples (mean ± SE) between core sampling sites.** Non-parametric test (Kruskal-Wallis One Way Analysis of Variance on Ranks) was performed with no differences between sites (H = 5.682, df = 5, p = 0.338).
(DOCX)

Table S3 **Comparison of temperature anomalies (calculated as cummulative degree days) between seasons for each core sampling site (cf.** **Fig. 1** **).** Non-parametric test (Wilcoxon rank test) was performed and test statistics are given (df = degrees of freedom, t = test-statistic and p = probability level, significance levels are: * p<0.05, ** p<0.01, *** p<0.001).
(DOCX)

Table S4 **Analysis of Variance (ANOVA) for temperature anomalies (calculated as cumulative degree days) during the dry season between core sampling sites (cf.** **Fig. 1** **).** Posthoc pair wise comparisons were performed via Tukey HSD-tests. (df = degrees of freedom; MS = means square; F = F-value; p = probability level, significance levels are: * p<0.05, ** p<0.01, *** p<0.001).
(DOCX)

Table S5 **Analysis of Variance (ANOVA) for bottom sediment grain size mean phi value between core sampling sites (cf.** **Fig. 1** **).** Posthoc pair wise comparisons were performed via Tukey HSD-tests. (df = degrees of freedom; MS = means square; F = F-value; p = probability level, significance levels are: * p<0.05, ** p<0.01, *** p<0.001).
(DOCX)

Table S6 **General linear models conducted for reef framework height as dependet variable with environmental parameters (temperature anomaly, grain size, sedimentation rate) as independent variable.** a) Framework height and environmental parameters derived from 6 core sampling sites and 2 additional sites (cf. Fig. 1). Parameters were quantified as: mean annual impact (Y) and maximum impact (max). Temperature anomaly maximum impact was calculated during the dry season in 2010 and 2011. (n = number of sites, r^2^ = regression coefficient, p = probability level, significance levels are: * p<0.05, ** p<0.01, *** p<0.001).
(DOCX)
