# Supplementary material for: Differential Impact of Monsoon and Large Amplitude Internal Waves on Coral Reef Development in the Andaman Sea
Source: PLoS One. 2012 Nov 28;7(11):e50207. doi: 10.1371/journal.pone.0050207 (PMC3509138; doi:10.1371/journal.pone.0050207)
Supplement: Table S1 — Cruise schedules and tasks. Time table for temperature logger, sediment trap and CTD deployments/exchanges at the core sampling sites (cf. Fig. 1). (DOCX) [file pone.0050207.s001.docx]

**Table S1. Cruise schedules and tasks.** Time table for temperature logger, sediment trap and CTD deployments/exchanges at the core sampling sites (cf. Fig. 1).

| **cruise** | **date** | **temperature logger exchange** | **sediment trap exchange** | **sediment cores** | **CTD** |
| --- | --- | --- | --- | --- | --- |
| Nov/Dec 2009 | 18.11-15.12 | start | start |  | x |
| Jan 2010 | 15.-25.01 | x | x | x |  |
| Feb/Mar 2010 | 26.02-20.03 | x | x | x | x |
| May 2010 | 19.-26.05. | x | x | x |  |
| Jul/Aug 2010 | 26.07-01.08 | x | x | x |  |
| Nov/Dec 2010 | 30.11-12.12 | x | x | x |  |
| Mar 2011 | 17.-27.03. | x | x | x |  |
| Aug 2011 | 23.-28.08 | x |  |  |  |
| Dec/Nov 2011 | 27.11-01.12 | x |  |  |  |
