# Supplementary material for: Differential Impact of Monsoon and Large Amplitude Internal Waves on Coral Reef Development in the Andaman Sea
Source: PLoS One. 2012 Nov 28;7(11):e50207. doi: 10.1371/journal.pone.0050207 (PMC3509138; doi:10.1371/journal.pone.0050207)
Supplement: Table S2 — Comparison of mud content (%) of bottom sediment samples (mean ± SE) between core sampling sites. Non-parametric test (Kruskal-Wallis One Way Analysis of Variance on Ranks) was performed with no differences between sites (H = 5.682, df = 5, p = 0.338). (DOCX) [file pone.0050207.s002.docx]

**Table S2. Comparison of mud content (%) of bottom sediment samples (mean ± SE) between core sampling sites.** Non-parametric test (Kruskal-Wallis One Way Analysis of Variance on Ranks) was performed with no differences between sites (H = 5.682, df = 5, p = 0.338).

| **sites** | **% weight mud** |
| --- | --- |
| Miang E | 4.64 ± 1.18 |
| Miang W | 3.65 ± 0.62 |
| Bon W | 2.75 ± 0.59 |
| Tachai W | 2.40 ± 0.44 |
| Surin W | 2.93 ± 0.57 |
| Racha W | 3.99 ± 1.14 |
