# Supplementary material for: Differential Impact of Monsoon and Large Amplitude Internal Waves on Coral Reef Development in the Andaman Sea
Source: PLoS One. 2012 Nov 28;7(11):e50207. doi: 10.1371/journal.pone.0050207 (PMC3509138; doi:10.1371/journal.pone.0050207)
Supplement: Table S3 — Comparison of temperature anomalies (calculated as cummulative degree days) between seasons for each core sampling site (cf. Fig. 1 ). Non-parametric test (Wilcoxon rank test) was performed and test statistics are given (df = degrees of freedom, t = test-statistic and p = probability level, significance levels are: * p<0.05, ** p<0.01, *** p<0.001). (DOCX) [file pone.0050207.s003.docx]

**Table S3. Comparison of temperature anomalies (calculated as cummulative degree days) between seasons for each core sampling site (cf. Fig. 1).** Non-parametric test (Wilcoxon rank test) was performed and test statistics are given (df = degrees of freedom, t = test-statistic and p = probability level, significance levels are: * p < 0.05, ** p < 0.01, *** p < 0.001).

| **sites** | **df** | **W** | **p** |
| --- | --- | --- | --- |
| Miang E | 10 | 0 | *** |
| Miang W | 10 | 0 | *** |
| Bon W | 10 | 0 | *** |
| Tachai W | 10 | 0 | *** |
| Surin W | 10 | 0 | *** |
| Racha W | 10 | 0 | *** |
