# Supplementary material for: Differential Impact of Monsoon and Large Amplitude Internal Waves on Coral Reef Development in the Andaman Sea
Source: PLoS One. 2012 Nov 28;7(11):e50207. doi: 10.1371/journal.pone.0050207 (PMC3509138; doi:10.1371/journal.pone.0050207)
Supplement: Table S4 — Analysis of Variance (ANOVA) for temperature anomalies (calculated as cumulative degree days) during the dry season between core sampling sites (cf. Fig. 1 ). Posthoc pair wise comparisons were performed via Tukey HSD-tests. (df = degrees of freedom; MS = means square; F = F-value; p = probability level, significance levels are: * p<0.05, ** p<0.01, *** p<0.001). (DOCX) [file pone.0050207.s004.docx]

**Table S4. Analysis of Variance (ANOVA) for temperature anomalies (calculated as cumulative degree days) during the dry season between core sampling sites (cf. Fig. 1).** Posthoc pair wise comparisons were performed via Tukey HSD-tests. (df = degrees of freedom; MS = means square; F = F-value; p = probability level, significance levels are: * p < 0.05, ** p < 0.01, *** p < 0.001).

| **Response** | **df** | **MS** | **F** | **p** |
| --- | --- | --- | --- | --- |
| Sites | 5 | 90.51 | 5.796 | *** |
| Residuals | 30 | 15.62 |  |  |
| TukeyHSD | Pairwise comparison | | | p |
| Miang E | > | Tachai W | | *** |
| Miang E | > | Miang W | | ** |
