# Supplementary material for: Differential Impact of Monsoon and Large Amplitude Internal Waves on Coral Reef Development in the Andaman Sea
Source: PLoS One. 2012 Nov 28;7(11):e50207. doi: 10.1371/journal.pone.0050207 (PMC3509138; doi:10.1371/journal.pone.0050207)
Supplement: Table S5 — Analysis of Variance (ANOVA) for bottom sediment grain size mean phi value between core sampling sites (cf. Fig. 1 ). Posthoc pair wise comparisons were performed via Tukey HSD-tests. (df = degrees of freedom; MS = means square; F = F-value; p = probability level, significance levels are: * p<0.05, ** p<0.01, *** p<0.001). (DOCX) [file pone.0050207.s005.docx]

**Table S5. Analysis of Variance (ANOVA) for bottom sediment grain size mean phi value between core sampling sites (cf. Fig. 1).** Posthoc pair wise comparisons were performed via Tukey HSD-tests. (df = degrees of freedom; MS = means square; F = F-value; p = probability level, significance levels are: * p < 0.05, ** p < 0.01, *** p < 0.001).

| **Response** | **df** | **MS** | **F** | **p** |
| --- | --- | --- | --- | --- |
| Sites | 5 | 1.321 | 5.91 | *** |
| Residuals | 99 | 0.223 |  |  |
| **TukeyHSD** | **Pairwise comparison** | | | **p** |
| Miang E | > | Racha W | | *** |
| Miang E | > | Tachai W | | ** |
| Miang E | > | Miang W, Bon W | | * |
| Surin W | > | Tachai W, Racha W | | * |
