# Supplementary material for: Differential Impact of Monsoon and Large Amplitude Internal Waves on Coral Reef Development in the Andaman Sea
Source: PLoS One. 2012 Nov 28;7(11):e50207. doi: 10.1371/journal.pone.0050207 (PMC3509138; doi:10.1371/journal.pone.0050207)
Supplement: Table S6 — General linear models conducted for reef framework height as dependet variable with environmental parameters (temperature anomaly, grain size, sedimentation rate) as independent variable. a) Framework height and environmental parameters derived from 6 core sampling sites and 2 additional sites (cf. Fig. 1). Parameters were quantified as: mean annual impact (Y) and maximum impact (max). Temperature anomaly maximum impact was calculated during the dry season in 2010 and 2011. (n = number of sites, r2 = regression coefficient, p = probability level, significance levels are: * p<0.05, ** p<0.01, *** p<0.001). (DOCX) [file pone.0050207.s006.docx]

**Table S6. General linear models conducted for reef framework height as dependet variable with environmental parameters (temperature anomaly, grain size, sedimentation rate) as independent variable.** a) Framework height and environmental parameters derived from 6 core sampling sites and 2 additional sites (cf. Fig. 1). Parameters were quantified as: mean annual impact (Y) and maximum impact (max). Temperature anomaly maximum impact was calculated during the dry season in 2010 and 2011. (n = number of sites, r^2^ = regression coefficient, p = probability level, significance levels are: * p < 0.05, ** p < 0.01, *** p < 0.001).

| **General linear model (Fig. 5 & 6):** | | | | | | |
| --- | --- | --- | --- | --- | --- | --- |
| **Frame~Environmnetal parameters of annual mean impact (Y) or max impact (max)** | | | | | | |
| **Environmental parameters** | **df** | **F-statistic** | **t-value (slope)** | **n** | **r^2^** | **p** |
| Temperature anomaly (Y) | 4 | 8.353 | 2.89 | 6 | 0.68 | 0.045 |
| Temperature anomaly (max) | 4 | 7.249 | 2.7 | 6 | 0.64 | 0.055 |
| Temperature anomaly (Y)^*)^ | 6 | 20.27 | 4.502 | 8 | 0.77 | 0.004 |
| Temperature anomaly (max 2010) ^*)^ | 6 | 18.48 | 4.298 | 8 | 0.75 | 0.005 |
| Grain size (Y) | 4 | 16.56 | 4.069 | 6 | 0.81 | 0.015 |
| Grain size (max) | 4 | 20 | 4.472 | 6 | 0.83 | 0.011 |
| Sedimentation (Y) | 4 | 1.512 | -1.229 | 6 | 0.27 | 0.286 |
| Sedimentation (max) | 4 | 1.572 | -1.254 | 6 | 0.28 | 0.278 |
| Temperature anomaly (max 2011) ^*)^ | 6 | 20.57 | 4.536 | 8 | 0.77 | 0.004 |
| Temperature anomaly (max) + Sedimentation (max): | 3 | 4.715 |  | 6 | 0.76 | 0.119 |
| Temperature anomaly (max) |  |  | 2.434 |  |  | 0.093 |
| Sedimentation max |  |  | -1.191 |  |  | 0.319 |
| Grain size max + Sedimentation max: | 3 | 12.21 |  | 6 | 0.89 | 0.036 |
| Grain size max |  |  | 4.085 |  |  | 0.027 |
| Sedimentation max |  |  | -1.254 |  |  | 0.299 |
| Temperature anomaly (max) + Grain size (max) | 3 | 54.84 |  | 6 | 0.97 | 0.004 |
| Temperature anomaly (max) |  |  | -3.973 |  |  | 0.029 |
| Grain size (max) |  |  | 6.088 |  |  | 0.009 |

^*)^ including core sampling sites plus additional sites from Schmidt et al. [16].
